# Seroprevalence and Determinants Associated with Mumps Antibodies after 20 Years of MMR Vaccination in Urban Area of Shanghai, China

**DOI:** 10.3390/ijerph15102089

**Published:** 2018-09-23

**Authors:** Hong Pang, Yibiao Zhou, Wensui Zhao, Qingwu Jiang

**Affiliations:** 1 Department of Epidemiology, School of Public Health, Fudan University, Shanghai 200032, China; 15111020009@fudan.edu.cn (H.P.); ybzhou@fudan.edu.cn (Y.Z); 2 Changning Center for Disease Control and Prevention, Shanghai 200051, China

**Keywords:** mumps, seroprevalence, vaccination, humoral immunity, MMR vaccine

## Abstract

A resurgence of the mumps epidemic in highly vaccinated populations has occurred in recent years in many countries. This study aimed to evaluate the seroprevalence to mumps in urban areas of Shanghai, where a measles-mumps-rubella (MMR) vaccination had been implemented for 20 years. Mumps IgG antibodies were tested in 2662 residual sera from all ages in an urban area of Shanghai. A linear regression method was performed to assess the persistence of mumps antibodies after MMR vaccination. A logistic regression method was used to analyze the variables associated with seronegative sera. The overall age- and gender-adjusted seroprevalence of mumps antibodies reached 90% (95% CI: 90.0–90.2). The antibody concentration declined significantly in the first eight years after the second dose of MMR. The multivariate analysis identified that males, age groups, especially 17–19 years and no dose of vaccination, as well as one dose of vaccination, as factors associated with an increased risk of seronegative sera. A high seroprevalence to mumps has been achieved in the urban areas of Shanghai. A declining antibody level of mumps after the second dose of MMR may put a potential risk of recurrence of mumps. The two-dose MMR vaccine schedule is superior to one-dose schedule for mumps control.

## 1. Introduction

Mumps is a common infectious disease in children, characterized by swelling of the salivary glands but can cause orchitis, pancreatitis, and aseptic meningitis. Mumps cases have been frequently reported in China in school-age children younger than 15 years, particularly aged 5–9 years [1,2]. The widespread use of mumps vaccines has decreased the incidence of the disease dramatically [3]. In 1996, Shanghai started measles-mumps-rubella (MMR) vaccination with a two-dose schedule, administered at 12–18 months and 4 years of age on a voluntary and self-paid basis. In 2008, the two-dose MMR schedule was incorporated into Shanghai immunization program and routinely offered free of charge to children aged 18 months and 4 years. Furthermore, during the measles catch-up supplemental immunization activities (SIAs) in China in 2010, one dose of MMR was implemented for students aged 6- to 14-year-old (born between September 1996 and August 2004) in Shanghai to achieve high levels of population immunity to measles. Three different mumps vaccine strains have been used in Shanghai: Jeryl-Lynn strain (Merk), RIT4385 strain (GlaxoSmithKline) and S79 strain (Shanghai Institute of Biological Products). With a high level of the two-dose MMR coverage, the incidence of mumps in Shanghai decreased gradually from 247.57 per 100,000 population in 1990 to 10.96 per 100,000 population in 2015, resulting in 95% decline in reported mumps incidence [4].

However, a resurgence of mumps epidemic in highly vaccinated adolescents and young adults has occurred in recent years in many countries. The most recent examples are the outbreaks in the United States and several European countries [5,6]. Some studies suggest that waning of vaccine-induced immunity is one of the causes of mumps outbreaks [7,8,9]. This sparked us to focus on humoral immunity to mumps in highly vaccinated population in Shanghai. Despite the high coverage rate of MMR, approximately average 3000 mumps cases per year were reported in Shanghai in the past decade. In addition to surveillance information of vaccine coverage and clinical notifications, serological surveillance has a valuable role in assessing the level of immunity to mumps following vaccination and may lead to the identification of subpopulations at an increased risk for mumps outbreaks. The aim of this study was to evaluate the seroprevalence of mumps antibodies in general population and identify the factors associated with seronegativity of mumps antibodies after 20 years of MMR vaccination in the urban area of Shanghai.

## 2. Materials and Methods

### 2.1. Study Site

Changning district, one of the 16 districts in Shanghai, is located in the west of central urban area and bordered the suburbs of Shanghai. It was selected as study site to represent the average level of development of Shanghai. In 2017, the total geographic area of Changning was 38 km^2^ and the total population was 720,000, of which 140,000 were migrants from other provinces according to the Bureau of Statistics of Shanghai.

### 2.2. Serological Survey 

A cross-sectional survey for mumps IgG antibodies was conducted from March to September in 2017. The serum samples from the younger age groups (≤19 years) were residual sera from children receiving a regular physical examination in five child health clinics and students receiving an annual physical examination in two designated diagnostic laboratories. The samples from the adult groups (≥20 years) were residual sera from the population receiving physical examinations in five hospitals of Changning district.

A total of 2662 serum samples from individuals aged 1 month to 96 years were collected and stratified into different age groups (0 to more than 60 years). The individuals with a history of MMR vaccination or a history of mumps within a month before blood sample collection were excluded. Information on mumps immunization of each child (<6 years) was retrospectively retrieved from the Shanghai Immunization Program Information System (SIPIS). Information on mumps immunization of each student (6–19 years) was retrospectively retrieved from immunization cards kept by the schools. Individuals aged ≥20 years were supposed to have no mumps immunization for Shanghai began to implement MMR vaccination after 1996. Demographic information, such as gender, age or date of birth, household registration (local or migrant status) were also collected through SIPIS, immunization cards or physical examination registration forms.

This study was approved by the medical ethics committee of the Changning Center for Disease Control and Prevention (CDC) (Code: 2017-1). The study was performed with research project CNKW2017Z02W, financed by Changning Commission of Science and Technology. Written informed consent was provided by individuals (aged ≥20 years) or parents of children (aged ≤19 years).

### 2.3. Laboratory Testing

Serum samples were collected and stored at −20 °C before being tested. Commercial enzyme-linked immunosorbent assay (ELISA) kits (Serion ELISA classic anti-mumps virus IgG, Institute Virion\Serion GmbH, Wurzburg, Germany, batch No.: SLF.CL) were used to detect and quantify human mumps IgG antibodies in serum. The IgG antibody concentration (U/mL) was calculated using the software from Serion and categorized as positive, equivocal, or negative using fixed cutoff values. Greater than 100 U/mL was considered positive, less than 30 U/mL was considered negative, and between 30 U/mL and 100 U/mL was considered equivocal. All the tests were carried out at the measles and rubella network laboratory in Changning CDC, which is a World Health Organization–certified reference laboratory for measles and rubella.

### 2.4. Statistical Analysis

Seroprevalence and geometric mean concentrations (GMCs) of mumps IgG antibodies were calculated, in addition to their 95% confidence intervals (95% CIs). Age and gender adjusted seroprevalence estimates were calculated for the whole population using the population of the sixth census in Changning in 2010 as the reference population.

Mumps vaccine coverage in age groups was calculated. Differences among seroprevalences by vaccination doses were assessed using the Pearson’s χ^2^ test.

A linear regression analysis was performed to assess the persistence of mumps antibodies after the first and second MMR vaccination. This analysis was restricted to individuals who had received their MMR vaccination(s) according to the Shanghai vaccination schedule. The once-vaccinated group included children aged 1–4 years, with one dose of MMR vaccinated at 1 year of age. The twice-vaccinated group included children aged 4–19 years, with two doses of MMR at 1 and 4 years-old respectively. The association between the antibody concentration and the time since first or second MMR vaccination was modeled. The optimal transformation for the variable time since vaccination was determined based on the Akaike information criterion (AIC).

A logistic regression analysis was performed to assess which variables were associated with a higher risk of being mumps antibody negative. Crude and adjusted odds ratios (aORs) were calculated in the univariable and multivariable analysis. Dichotomizations were performed by combining negative and equivocal sera results in the negative category.

The data were analyzed with Stata 12.0 (Statacorp LP, College Station, TX, USA), with a two-sided significance level of *p* < 0.05.

## 3. Results

### 3.1. Seroprevalence and GMCs of Mumps Antibodies

**Figure 1:**
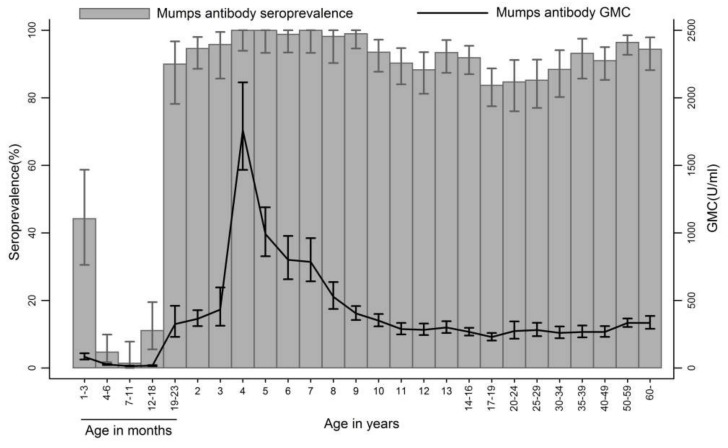
Age specific seroprevalence and GMC of mumps antibody. The error bars represent the 95% CI.

The serum samples from 2662 individuals (51.2% males) were tested. Of these, 2184 (82.0%), 385 (14.5%), and 93 (3.5%) were sera positive, negative, and equivocal to mumps antibodies, respectively. The both age- and gender-adjusted seropositivity was 90.1% (95% CI: 90.0–90.2), while the age-adjusted seropositivity among males and females was 89.2% (95% CI: 89.1–89.3) and 90.9% (95% CI: 90.8–91.0), respectively. The seroprevalence of maternal mumps antibodies declined rapidly to 1% (95% CI: 0–8) at age 7–11 months (Table 1 and Figure 1). After the first MMR (MMR1) vaccination at age 18 months, the seroprevalence increased rapidly in the subsequent age groups and reached 90% (95% CI: 78–97) at age 19–23 months. After the second MMR (MMR2) vaccination at age 4 years, the seroprevalence reached 100% at age 4 years and maintained at high levels during the first 5 years after MMR2. Then the seroprevalence declined gradually to 88% (95% CI: 83–94) at age 12 years. The seroprevalence in the cohorts covered by MMR catch-up SIAs increased at age 13–16 years. Among individuals aged ≥20 years, whose immunity resulted more likely from natural infection, the seroprevalence gradually increased from 85% in the age group of 20–24 years to 94% in the oldest age group.

The overall GMC of mumps antibodies of samples was 245 U/mL (95% CI: 233–257). The GMCs among age groups generally followed the trend of age-specific seroprevalence. The highest GMC value (1761 U/mL) occurred at age 4 years, but it declined rapidly to level similar to pre-MMR2 values at age 9 years. The GMCs in individuals aged ≥20 years were maintained at a stable level, which ranged between 260 U/mL and 334 U/mL.

### 3.2. Vaccination Coverage and Mumps Seroprevalence

**Figure 2:**
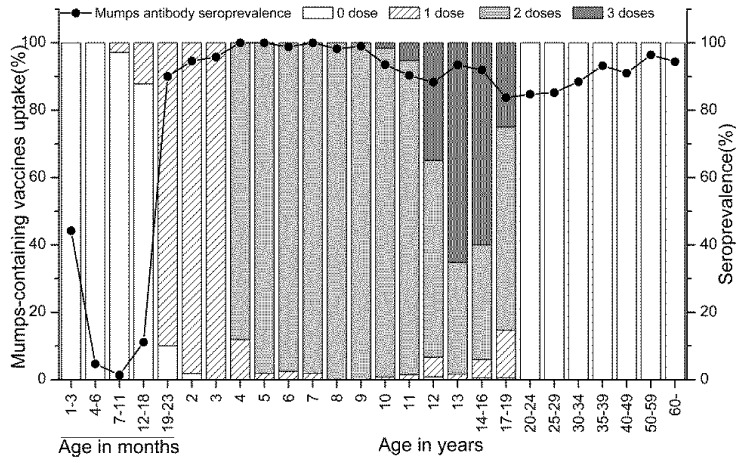
Doses of mumps-containing vaccines and mumps antibody seroprevalence. No (0) dose in ≥20 years old: no information about the vaccination status had been obtained and that some of these person might have been vaccinated.

The coverage rate of MMR1 for children aged 19–23 months was 90%, and it reached 98% at age 2 years (Figure 2). The coverage rate of MMR2 for children aged 4 years was 88%, and it also reached 98% at age 5 years. 11 children aged ≤18 months and 37 children aged 10–19 years had been vaccinated with one dose of monovalent mumps vaccine or bivalent measles–mumps vaccine, which were offered to children aged ≥8 months.

Children aged 12–19 years (whose serum samples were collected on March 2017) were the MMR target population in measles catch-up SIAs in September 2010. Among 610 individuals aged 12–19 years, additional MMR vaccines were administered to 417 individuals, including 225 (54%) third dose of MMR (MMR3), 169 (40%) MMR2 and 23 (6%) MMR1. Among the 610 individuals, mumps seroprevalence of MMR catch-up SIAs covered individuals was higher than that of not covered individuals (92% (95% CI: 89–94) and 83% (95% CI: 77–88), respectively, χ^2^ = 9.0448, *p* < 0.01) and mumps antibody seroprevalence of three-dose-vaccinated individuals was higher than that of two- and one-dose-vaccinated individuals (94% (95% CI: 90–96) and 85% (95% CI: 81–89), respectively, χ^2^ = 11.982, *p* < 0.01).

### 3.3. Waning Immunity of Mumps

**Figure 3:**
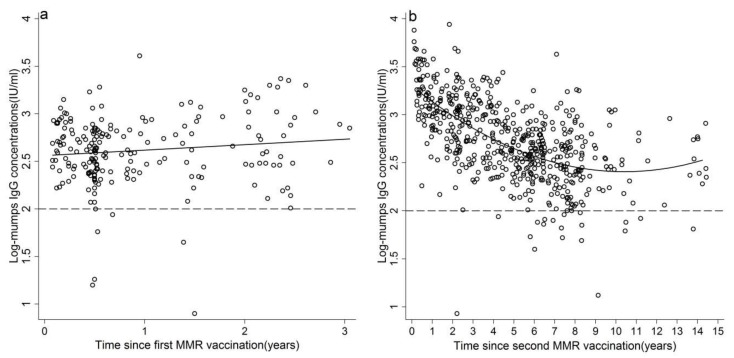
Mumps antibody persistence after first (**a**) and second (**b**) MMR vaccination. The fitted model (line) and cut-off for seropositivity (dash line).

After MMR1 vaccination, the antibody concentration did not decrease in 3 years (F = 3.05, *p* = 0.08, Figure 3). After MMR2 vaccination, the antibody concentration declined significantly in the first 8 years (F = 168.51, *p* < 0.01) and remained constant in the years thereafter.

### 3.4. Determinants for Seronegativity of Mumps Antibodies

The multivariate analysis showed that males (aOR = 1.4, 95% CI: 1.0–1.8), age subgroups 0–18 months (aOR = 165.6, 95% CI: 86.7–316.4), 10–12 years (aOR = 2.2, 95% CI: 1.1–4.2), 17–19 years (aOR = 4.2, 95% CI: 2.1–8.3), 20–29 years (aOR = 3.8, 95% CI: 1.9–7.5), 30–39 years (aOR = 2.2, 95% CI: 1.0–4.7), 40–49 years (aOR = 2.3, 95% CI: 1.0–5.0) and no vaccination (aOR = 139.8, 95% CI: 38.3–510.9), one-dose vaccination (aOR = 3.9, 95% CI: 1.7–9.0) were associated with an increased risk of being seronegative of mumps antibodies. No significant difference was found in risk of being seronegtive between local residents and migrants (aOR = 1.1, 95% CI: 0.8–1.5) (Table 2).

## 4. Discussion

This study presents the mumps-specific antibody levels of a population-based serosurveillance study performed in 2017, 20 years after the introduction of MMR in the urban area of Shanghai. The overall standardized seroprevalence in this present study reached 90.1% (95% CI: 90.0–90.2). According to two mathematical models, the herd immunity threshold of 85%–90% would be needed to prevent circulation of the mumps virus [10,11] and one study estimated the threshold of 88%–92% [12], the MMR vaccination had established a herd immunity in the population of Shanghai, which might block the spread of mumps.

Although a high mumps seroprevalence in the population was observed, some results were still worth noting. The two-dose MMR immunization in Shanghai clearly induced a sharp increase in mumps antibodies concentration in the corresponding age groups. But the antibody levels declined rapidly with time since the second dose. An exponential decrease of Log-transformed antibody concentration was observed in the first 8 years after MMR2. The seroprevalence of mumps antibodies also decreased to 83% (95% CI: 77–88) in individuals aged 12–19 years not covered by MMR catch-up SIAs, which was under the threshold of herd immunity. Several serological studies have shown mumps antibody levels decline as time from the second vaccination increases [13,14,15,16,17]. In the absence of mumps virus circulation and natural boosting after widespread use of MMR, vaccine-induced antibodies may wane over time. On the other hand, in view of the measles vaccination, the second dose of measles containing vaccine was not meant as a booster dose, but as a catch-up for susceptible subjects, who either missed or did not respond to the first dose [18]. So the second dose of MMR produced a transient rise in mumps antibody levels instead of a sustained high level. Decreasing antibody levels with increasing time after receipt of MMR2 are consistent with observations of mumps recurrence among highly vaccinated adolescents and young adults in many countries in recent years. Young adults in close-contact settings, such as people on college campuses have been mainly affected by mumps recurrence [19]. In Shanghai, the highest incidence of mumps was still reported among children of primary school age and no mumps epidemic among adolescents or young adults was reported in the vaccine era [4]. Reasons might be: (1) The immunity of individuals of previously MMR catch-up SIAs covered cohorts may buffer transmission and delay breakthrough epidemics. (2) People aged ≥20 years in Shanghai were mostly born in the pre-vaccine era. They might develop immunity through natural infection in their childhood. (3) With sustaining decline in mumps incidence and complication rate in vaccine era, expectations that mumps appears in pediatric rather than adult population may bias disease reporting, especially when mumps surveillance is based on clinical diagnosis. The decline seroprevalence of mumps since MMR2 in our study suggests when the population covered by MMR catch-up SIAs and born before the introduction of MMR vaccination are gradually replaced by the population whose protection comes only through vaccination, it is also possible that the recurrence of mumps cases in highly vaccinated adolescents and young adults. The immunity of population to mumps at and after the age of 12 years might determine the epidemic characteristics of mumps in the vaccine era.

The waning immunity and mumps outbreaks among two-dose MMR recipients have raised discussion regarding the administration of a third dose of mumps-containing vaccine as a booster later in life. However, a recommendation for routine use of a third dose was not made as data remain insufficient. Three epidemiologic studies conducted in schools and a university, suggested effectiveness of using MMR3 as public health intervention for mumps outbreak control [20,21,22]. Two immunogenicity studies administered MMR3 to subjects aged 18-28 years, demonstrated a significant increase in mumps antibodies one month after vaccination; however antibody titers returned to near-baseline levels a year later [23,24]. In light of the available evidence, a third dose of mumps containing vaccine is recommended in the US only to persons at increased risk for acquiring mumps because of an outbreak [25]. Our results showed that three-dose recipients had higher seroprevalence than students with ≤2 doses even after 6 years. If the MMR3 can provide a sustained elevation in mumps seroprevalence, it may extend mumps protection to older ages and be beneficial for certain at-risk groups. So our study suggests that if the burden of mumps shifts to adolescents or young adults in vaccine era, MMR3 could be administered to them to improve protection against mumps. Taking into account mortality and disease burden, vaccination against mumps has “followed” the strategy of vaccination against measles and rubella in the number of doses and schedule of administration [26]. In Shanghai, an additional dose of measles vaccine is also administered to college students of grade one regardless their vaccination history. The MMR instead of measles vaccine could be administered to college students to maintain immune protection or as an outbreak control measure.

Our results still showed that gender, age and number of doses for vaccination were associated with seronegativity of mumps antibodies. A higher seronegativity was observed in males than females. A mumps seroprevalence study in Taiwan reported a trend toward higher seronegativity in males compared to females at 2–20 years of age after two-dose childhood mumps-containing vaccine implemented for >20 years [13]. A cross-sectional population-based serosurveillance study in the Netherlands also reported a significant higher seronegativity of mumps antibodies in males [14]. Females exhibit higher rates of seropositivity for most antigens including mumps virus compared to males [27]. The higher seronegativity in males was also supported by an epidemiology study in China that the incidence of mumps in males was higher than that in females during 2004 to 2013 [28]. Except for children targeted by MMR1 (aged 19–36 months), MMR2 (aged 4–9 years) and MMR catch-up SIAs (aged 13–16 years), higher seronegativity rates of mumps antibodies were observed in young individuals than in elderly (aged ≥50 years). This suggested a longer duration of seropositivity induced by natural infection compared to that generated by immunization. Except for age group 0-18 months, the higher risk of seronegative sera in age group 17–19 years was noteworthy. Waning immunity to mumps with a period of time since an MMR vaccination seemed to be the most plausible explanation. The seronegativity of the no (0) dose and one-dose vaccinated population were significantly higher than that of those with a history of two or three doses, indicating that the two-dose MMR vaccine schedule provides better humoral protection against mumps than the one-dose schedule. A serosurvey conducted on students aged 17–23 years has shown that the seropositivity of mumps antibodies and GMTs were significantly higher in two-dose MMR vaccine group than in one-dose group [29]. Accumulated global experience had demonstrated that the prevention of mumps requires sustained high levels of immunization coverage and more than one dose of the vaccine [26]. In 2008, MMR was introduced into the Chinese national routine immunization program, and one dose of MMR was routinely administered at 18–24 months. But the incidence of mumps in China was not significantly reduced in 2010–2013 [28]. Some more developed cities in China, such as Shanghai, Beijing, and Tianjin, which introduced two doses of MMR into routine immunization program, had a better controlling effect on the incidence of mumps [28]. No difference in seronegativity was found between the local and migrant population, indicating that local and migrant children had the same opportunity for vaccination.

The strengths of our study are worth mentioning. To the best of our knowledge, this study is the first to report the humoral immunity to mumps under the two-dose MMR schedule in China. We studied a wide age group that allowed for the analysis of different opportunities for natural or artificial exposure to the mumps virus. We obtained a clear mumps vaccination status of individuals aged ≤19 years old from the immunization information system or immunization cards, which allowed us to analyze the relationship between mumps seroprevalence and vaccine doses.

Our study had some limitations. First, as opposed to randomized samples, convenient samples of residual serum were used to evaluate the seroepidemiology of mumps among the population. Whether convenient samples can be used to evaluate the serosurvey of mumps needs to be verified in the future. The same sera of our study can be used to determine antibodies against other vaccine-preventable diseases, such as measles and rubella. So the high coverage of MMR and high level of mumps seropositivity in children in our study can be supported. Second, MMR immunization coverage rates among the participants aged ≥20 years was unknown in our study, but the coverage was very likely to be quite lower than that among the individuals aged <20 years. Third, the clinical mumps cases reported to the Changning CDC were not routinely confirmed by laboratory methods, we were therefore unable to correlate the seroepidemiology data with the age and MMR doses distributions of confirmed mumps cases.

## 5. Conclusions

In conclusion, a high seroprevalence of mumps antibodies has been achieved in urban areas of Shanghai after 20 years of the introduction of MMR vaccination. A two-dose MMR vaccine schedule provides better humoral immunity against mumps than one-dose schedule. A declining antibody level to mumps was evident after the second dose of MMR, signaling a potential risk of recurrence of mumps in people aged more than 12 years. A third dose of MMR may help control the recurrence of mumps among adolescents and younger adults.

## Figures and Tables

**Table 1:** Age- and gender-specific mumps IgG seroprevalence (%) and GMC (U/mL).

Age	Total			Males			Females		
	n	% (95% CI)	U/mL (95% CI)	n	% (95% CI)	U/mL (95% CI)	n	% (95% CI)	U/mL (95% CI)
1–3 months	52	44 (30–59)	82 (62–109)	23	35 (16–57)	79 (50–126)	29	52 (33–71)	85 (59–121)
4–6 months	128	5 (2–10)	25 (21–29)	65	6 (2–15)	26 (21–32)	63	3 (0–11)	24 (19–30)
7–11 months	69	1 (0–8)	14 (12–17)	38	0 (0-9)	14 (12–16)	31	3 (0–17)	15 (12–20)
12–18 months	90	11 (5–19)	17 (14–21)	47	13 (5–26)	19 (14–26)	43	9 (3–22)	16 (12–21)
19–23 months	50	90 (78–97)	325 (230–460)	31	90 (74–98)	364 (236–562)	19	89 (67–99)	271 (145–505)
2 years	111	95 (89–98)	364 (310–427)	52	92 (81–98)	329 (262–414)	59	97 (88–100)	398 (317–499)
3 years	48	96 (86–99)	432 (313–596)	23	96 (78–100)	368 (218–621)	25	96 (80–100)	500 (328–761)
4 years	59	100 (94–100)	1761 (1467–2114)	26	100 (87–100)	1731 (1344–2229)	33	100 (89-100)	1785 (1361–2341)
5 years	53	100 (93–100)	992 (827–1190)	29	100 (88–100)	1057 (805–1388)	24	100 (86-100)	918 (716–1178)
6 years	82	99 (93–100)	801 (656–978)	52	100 (93–100)	772 (634–939)	30	97 (83–100)	855 (549–1333)
7 years	53	100 (93–100)	785 (641–961)	33	100 (89–100)	748 (580–965)	20	100 (83–100)	849 (591–1220)
8 years	55	98 (90–100)	527 (437–636)	29	100 (88–100)	480 (375–614)	26	96 (80–100)	586 (434–790)
9 years	100	99 (95–100)	404 (355–459)	58	98 (91–100)	387 (317–472)	42	100 (92–100)	428 (374–491)
10 years	124	94 (88–97)	351 (308–399)	66	91 (81–97)	321 (269–382)	58	97 (88–100)	388 (320–471)
11 years	134	90 (84–95)	288 (249–333)	83	90 (82–96)	271 (232–317)	51	90 (79–97)	316 (235–426)
12 years	120	88 (81–93)	283 (244–329)	68	85 (75–93)	260 (211–321)	52	92 (81–98)	316 (256–389)
13 years	121	93 (87–97)	300 (259–346)	53	89 (77–96)	295 (224–388)	68	97 (90–100)	303 (261–353)
14-16 years	185	92 (87–95)	267 (240–297)	95	88 (80–94)	247 (212–288)	90	96 (89–99)	289 (250–335)
17–19 years	184	84 (78–89)	229 (202–259)	101	83 (74–90)	217 (186–254)	83	84 (75–91)	244 (199–299)
20–24 years	98	85 (76–91)	273 (217–344)	56	84 (72–92)	250 (181–346)	42	85 (71–95)	308 (220–430)
25–29 years	108	85 (77–91)	281 (236–334)	55	84 (71–92)	248 (199–311)	53	87 (75–95)	319 (243–419)
30–34 years	95	88 (80–94)	260 (220–307)	50	88 (76–95)	231 (185–288)	45	89 (76–96)	296 (230–382)
35–39 years	88	93 (86–97)	266 (226–314)	45	91 (79–96)	243 (190–312)	43	95 (84–99)	293 (235–366)
40–49 years	155	91 (85–95)	267 (230–310)	50	94 (83–99)	289 (229–364)	105	90 (82–95)	257 (212–312)
50–59 years	193	96 (93–99)	333 (304–366)	91	97 (91–99)	321 (280-369)	102	96 (90–99)	345 (303–392)
60 and above	107	94 (88–98)	334 (290–385)	45	91 (79–98)	292 (224–381)	62	97 (89–100)	368 (316–429)
Total	2662	82 (81–83)	245 (233–257)	1233	81 (79–83)	234 (219–251)	1199	83 (81–85)	257 (239–275)

**Table 2:** Potential factors for seronegativity of mumps antibody with corresponding crude and adjusted odds ratios (ORs).

Factors	*n*	Seronegativity (%, 95% CI)	Univariate Analysis		Multivariate Analysis	
			Crude OR (95%CI)	*p* Value	Adjusted OR * (95% CI)	*p* Value
**Sex**				0.128		
Male	1364	19.1 (17.0–21.2)	1.2 (0.96–1.4)		1.4 (1.0–1.8)	0.026
Female	1298	16.8 (14.8–18.9)	Reference		Reference	
**Age (years)**				<0.0001		
0–18 months	339	88.2 (84.3–91.4)	165.0 (86.5–315.0)		165.6 (86.7–316.4)	<0.0001
19–36 months	209	6.2 (3.4–10.4)	1.5 (0.7–3.2)		1.4 (0.7–3.2)	0.366
4–9	402	0.7 (0.2–2.2)	0.2 (0.0–0.6)		0.2 (0.0–0.6)	0.005
10–12	378	9.3 (6.5–12.4)	2.3 (1.2–4.3)		2.2 (1.1–4.2)	0.021
13–16	306	7.5 (4.8–11.1)	1.8 (0.9–3.6)		1.8 (0.9–3.6)	0.107
17–19	184	16.3 (11.3–22.5)	4.3 (2.2–8.5)		4.2 (2.1–8.3)	<0.0001
20–29	206	15.0 (10.5–20.7)	3.9 (2.0–7.7)		3.8 (1.9–7.5)	<0.0001
30–39	183	9.3 (5.5–14.5)	2.3 (1.1–4.8)		2.2 (1.0–4.7)	0.037
40–49	155	9.0 (5.0–14.7)	2.2 (1.0–4.8)		2.3 (1.0–5.0)	0.038
≥50	300	4.3 (2.3–7.3)	Reference		Reference	
**Household registration**				0.016		
Local	1449	19.6 (17.6–21.7)	1.3 (1.0–1.6)		1.1 (0.8–1.5)	0.435
Migrant	1213	16.0 (14.0–18.2)	Reference		Reference	
**Number of vaccinations**				<0.0001		
0 dose	1180	32.0 (29.4–34.8)	7.0 (4.3–11.5)		139.8 (38.3–510.9)	<0.0001
1 dose	274	8.8 (5.7–12.8)	1.4 (0.8–2.7)		3.9 (1.7–9.0)	0.001
2 doses	921	6.3 (4.8–8.1)	1.0 (0.6–1.7)		1.7 (0.9–3.0)	0.102
3 doses	287	6.3 (3.8–9.7)	Reference		Reference	

* Adjusted for age and gender.
